# When Stroke Leads to Hydrocephalus: A Case of Unilateral Ventricular Enlargement

**DOI:** 10.7759/cureus.110014

**Published:** 2026-05-31

**Authors:** Venkata Kaumudi Ayaluri, Harshitha Reddy, Anurag Mantri Vera Sai

**Affiliations:** 1 Accident and Emergency, Bronglais General Hospital, Aberystwyth, GBR; 2 Internal Medicine, Jawaharlal Nehru Medical College, Wardha, IND; 3 Public Health, York St John University, London, GBR

**Keywords:** foramen of monro, gliosis, ischemic stroke, obstructive hydrocephalus, recurrent stroke, unilateral hydrocephalus

## Abstract

Unilateral hydrocephalus is a rare form of ventricular enlargement, typically resulting from obstruction of the cerebrospinal fluid (CSF) flow at the foramen of Monro. Unilateral hydrocephalus is rarely reported as a complication of recurrent ischemic stroke. While tumours, cysts, and infections are common etiologies, vascular causes are rarely reported. We present a 58-year-old female patient who presented to the emergency room in an irritable state. She also had acute right-sided weakness of upper and lower limbs along with aphasia. Neuroimaging revealed Gliosis with encephalomalacic changes in the left fronto-temporal lobes and left capsuloganglionic region, causing ipsilateral dilatation of the left lateral ventricle. The likely mechanism involved post-stroke gliosis and obstruction of CSF pathways. This case highlights the importance of recognising atypical causes of hydrocephalus in stroke patients, especially when clinical deterioration is disproportionate to infarct size. Need to prevent irreversible neurological damage in this condition, so diagnosis and prompt treatment are required.

## Introduction

Abnormal accumulation of cerebrospinal fluid (CSF) within the ventricular system of the brain is known as Hydrocephalus. It is characterised by ventricular dilatation and, in turn, elevated intracranial pressure [1]. In general, there are two forms of hydrocephalus: communicative and non-communicating (obstructive). When there is impaired absorption of CSF by the arachnoid granulations, it is termed communicating hydrocephalus. However, focal occlusion within the ventricular system causes non-communicating hydrocephalus, which results in upstream ventricular enlargement [1]. Unilateral hydrocephalus is a rare and unique subtype of obstructive hydrocephalus that is distinguished from other types of hydrocephalus by the dilation of a single lateral ventricle [2]. The narrow canal that connects each lateral ventricle to the third ventricle is the foramen of Monro, and obstruction at this level occurs in this condition. Common causes of unilateral hydrocephalus include colloid cysts, intraventricular tumors, congenital anomalies, inflammatory processes, and infections such as neurocysticercosis or tuberculoma [2]. However, in contrast to these well-recognized etiologies, vascular causes, particularly stroke, are rarely implicated.

The majority of stroke cases are ischemic stroke, which continues to be one of the world's leading causes of morbidity and death. Hydrocephalus is more commonly associated with hemorrhagic stroke, particularly in cases of intraventricular hemorrhage or subarachnoid hemorrhage, where blood products obstruct CSF pathways or impair absorption [3]. In contrast, ischemic stroke is only rarely associated with hydrocephalus, and when it occurs, it is typically in the setting of large territorial infarctions causing mass effect and secondary ventricular compression. An ischemic stroke leading to hydrocephalus is an uncommon phenomenon. Infarcts in the basal ganglia, thalamus, or periventricular white matter lie in proximity to CSF pathways, and stroke in these regions can cause obstruction at the foramina of Monro. These channels may become distorted or narrowed over time due to post-infarct gliosis, scarring, and tissue remodeling, which would gradually limit CSF flow [4].

Neuroimaging is essential in the diagnosis of this condition. Magnetic resonance imaging (MRI) allows detailed visualisation of the ventricular anatomy and associated parenchymal changes. The degree of symptoms and the underlying blockage mechanism determine how unilateral hydrocephalus is managed. In mild cases, conservative measures can be used, but definitive treatment requires surgical intervention. In this report, we describe a rare case of unilateral obstructive hydrocephalus that developed as a post-stroke complication [5].

## Case presentation

A 58-year-old female presented to the emergency department with complaints of sudden onset weakness in both upper and lower limbs on the right side, aphasia, and altered sensorium. There was no history of fever, seizures, or recent head trauma. The patient had a significant history of hypertension. Her neurological history includes two prior strokes over the past three years, resulting in residual right-sided weakness. She was on regular antihypertensive medications, antiplatelet therapy, and physiotherapy.

On examination, the patient was drowsy but arousable, with a Glasgow Coma Scale (GCS) score of 12/15 (E3V4M5). Her Blood pressure on arrival was 230/120 mmHg, which was promptly addressed by giving her labetolol. Neurological examination revealed right-sided hemiparesis with a motor strength of 1/5 in both the upper and lower limbs, along with exaggerated deep tendon reflexes and an extensor plantar response on the right side. Pupils were equal and reactive to light, and no cranial nerve deficits were noted. There were no signs of meningeal irritation.

Magnetic resonance imaging (MRI) of the brain was performed, which revealed Gliosis with encephalomalacic changes noted in the left fronto-temporal lobes and the left capsuloganglionic region, and also causing ipsilateral dilatation of the left lateral ventricle (Figures 1, 2, 3).

**Figure 1:**
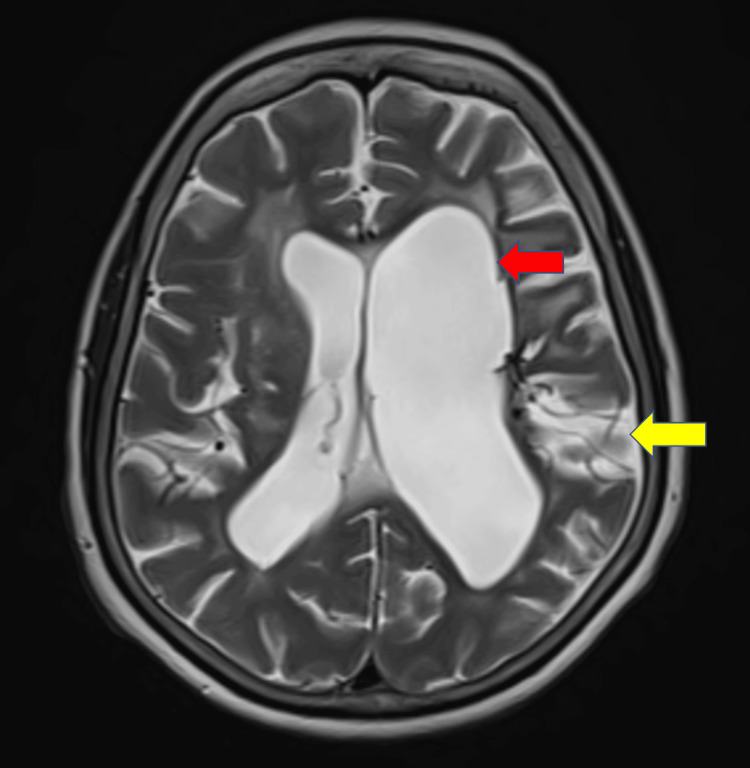
MRI brain T2W image showing left lateral ventricle enlarged (Red arrow) than the right and Gliosis of the left temporal region (Yellow arrow)

**Figure 2:**
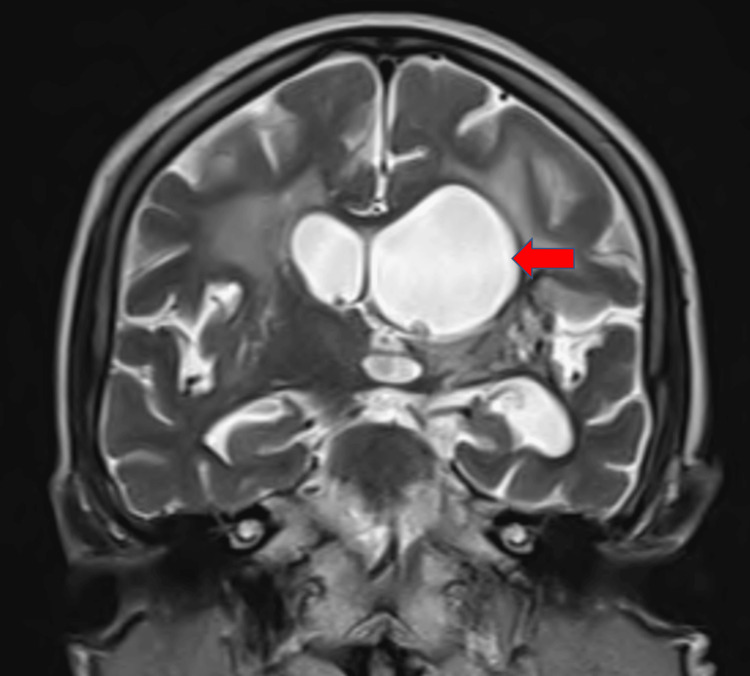
MRI brain T2W image coronal view showing left lateral ventricle enlargement (Red arrow)

**Figure 3:**
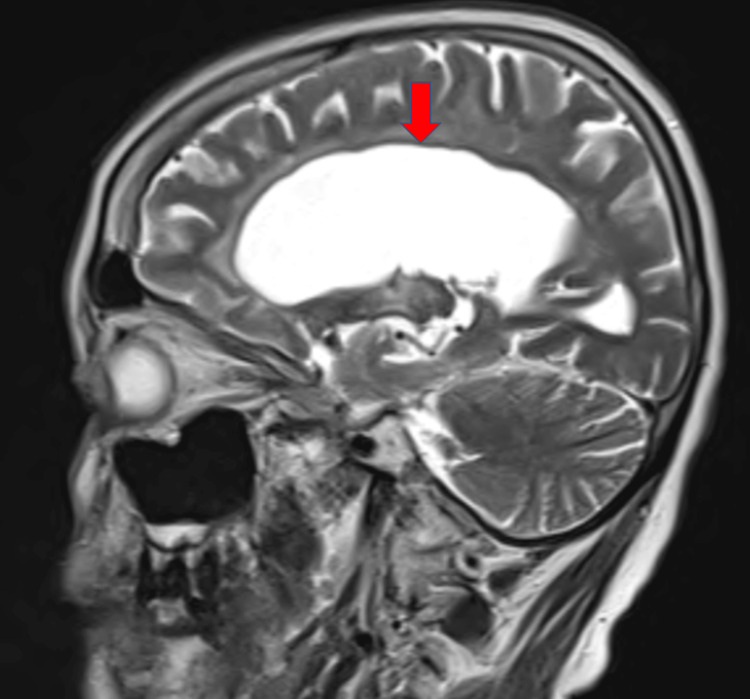
MRI brain T2W image sagittal view showing left lateral ventricle enlargement (Red arrow)

Based on the clinical and radiological findings, a diagnosis of unilateral obstructive hydrocephalus secondary to gliotic obstruction of the foramen of Monro following recurrent ischemic strokes was made. The diagnosis was presumptive and based on the combination of unilateral ventricular enlargement, asymmetric dilatation relative to the degree of parenchymal loss, and the overall radiologic pattern suggestive of impaired CSF outflow.

Treatment of the patient included anti-hypertensives such as labetolol, telmisartan, benedipin, and prazosin with regular Blood pressure monitoring so as not to cause secondary infarction on the penumbra region. Unilateral Hydrocephalus was managed conservatively with head elevation, empirical osmotic therapy (intravenous mannitol) was started, and close neurological monitoring. She was on other medications such as antiplatelet therapy, anticoagulants, and physiotherapy. She improved gradually and was discharged as her GCS was 15/15. She was advised to undergo neurosurgical intervention but she and her relatives were not willing for it, so she was being managed conservatively with frequent neurological monitoring.

## Discussion

Hydrocephalus is a neurological condition characterized by the abnormal accumulation of cerebrospinal fluid (CSF) within the ventricular system of the brain, resulting in ventricular dilatation and, in many cases, elevated intracranial pressure [1]. Traditionally, hydrocephalus is broadly classified into communicating and non-communicating (obstructive) types, depending on whether there is a mechanical blockage within the CSF pathways. Communicating hydrocephalus occurs when CSF flow remains unobstructed within the ventricular system, but absorption at the level of the arachnoid granulations is impaired, whereas non-communicating hydrocephalus arises due to focal obstruction within the ventricular system, leading to upstream ventricular enlargement [1]. Among the various forms of hydrocephalus, unilateral hydrocephalus represents a rare and distinct subtype of obstructive hydrocephalus, characterized by the dilatation of a single lateral ventricle. This condition most commonly results from obstruction at the level of the foramen of Monro, the narrow channel connecting each lateral ventricle to the third ventricle. The foramen of Monro is particularly vulnerable to obstruction due to its small caliber and its anatomical location [2]. Common causes of unilateral hydrocephalus include colloid cysts, intraventricular tumors such as central neurocytomas and subependymomas, intraventricular hemorrhage, inflammatory processes such as ventriculitis, and congenital anomalies leading to stenosis or atresia of the foramen, and infections such as neurocysticercosis or tuberculoma [2]. However, in contrast to these well-recognized etiologies, vascular causes, particularly stroke, are rarely implicated.

Acute ischemic stroke is a leading cause of morbidity and mortality worldwide, with well-documented complications including cerebral edema, hemorrhagic transformation, seizures, and increased intracranial pressure. Among these, cerebral edema plays a critical role in early neurological deterioration. Following ischemic injury, a cascade of pathophysiological events leads to the development of cytotoxic edema, characterized by intracellular swelling due to failure of ionic pumps, followed by vasogenic edema resulting from disruption of the blood-brain barrier [3,4]. After an ischemic stroke, edema usually develops in the first few days and peaks between three and five days. The resulting mass effect can be significant in large infarcts, especially those involving deep brain areas. While motor impairments, seizures, cognitive impairment, and speech abnormalities are among the well-known neurological after effects of stroke, few secondary problems involving CSF dynamics are less common [3,4].

Usually, hemorrhagic stroke leads to Hydrocephalus due to intraventricular hemorrhage or subarachnoid hemorrhage, where blood products obstruct CSF pathways or impair absorption. On the other hand, hydrocephalus is infrequently linked to ischemic stroke, and when it does, it usually happens in the context of extensive territorial infarctions that result in secondary ventricular compression and mass impact. Pathogenesis in ischemic stroke involves structural and inflammatory changes rather than acute obstruction, like in hemorrhagic stroke. Infarcts involving the basal ganglia, thalamus, or periventricular white matter regions lie in proximity to CSF pathways, including the foramen of Monro. These channels may become distorted or narrowed over time due to post-infarct gliosis, scarring, and tissue remodeling, which would gradually limit CSF flow [3,4]. 

In particular, unilateral hydrocephalus poses special diagnostic and treatment challenges. Since only one lateral ventricle is impacted, the condition may appear mild on initial imaging and be missed if ventricular symmetry is not carefully considered. Acute ischemic stroke can cause unilateral hydrocephalus; the main neurological impairments may take precedence [5,6]. Patients may develop a sudden or progressive decline in consciousness, increasing headaches, projectile vomiting, gait abnormalities, and bowel and bladder involvement. These symptoms may be misdiagnosed as an increase in the size of the infarct itself rather than post-stroke complications such as hydrocephalus. There is an overlap in the clinical features, which might lead to a delay in diagnosis and treatment. Consequently, a strong index of suspicion is necessary, especially in patients who show clinical deterioration that is out of proportion to the first stroke's expected course [6].

When diagnosing unilateral hydrocephalus, neuroimaging is essential. Depending on the severity, non-contrast computed tomography (CT), which is frequently the first-line modality, may show asymmetric dilatation of one lateral ventricle along with a mass effect and midline shift. The ability to see ventricular morphology, CSF flow dynamics, and related parenchymal alterations in great detail makes magnetic resonance imaging (MRI) especially useful. Asymmetric enlargement of a lateral ventricle, periventricular hyperintensities suggesting transependymal CSF seepage, and signs of previous infarcts in nearby areas are typical observations. In some cases, careful evaluation may reveal narrowing or obstruction at the level of the foramen of Monro, although this may be subtle and requires high-resolution imaging and multiplanar assessment [6,7]. A key radiologic differential diagnosis in this case was ex vacuo ventricular dilatation secondary to chronic encephalomalacia and gliosis following prior ischemic injury. However, the marked asymmetry of ventricular enlargement, relative disproportion to the degree of parenchymal loss, and imaging features suggestive of impaired CSF drainage at the foramen of Monro favored a component of unilateral obstructive hydrocephalus rather than solely compensatory ventricular expansion.

In the present case, recurrent ischemic injury involving the periventricular and deep cerebral regions was considered a possible contributor to impaired CSF drainage and subsequent unilateral ventricular enlargement. Unlike the more common causes of unilateral hydrocephalus, such as tumors, cysts, hemorrhage, or congenital stenosis, the radiologic findings in this patient suggested a more complex mechanism related to chronic post-ischemic structural changes. Gliosis, tissue remodeling, and encephalomalacic changes adjacent to the ventricular system may have contributed to the distortion or narrowing of the foramen of Monro, resulting in impaired CSF flow. Although significant gliosis and parenchymal loss were present, the marked asymmetry of ventricular enlargement and the apparent disproportion between ventricular size and the degree of tissue loss favoured a possible obstructive component rather than purely compensatory ventricular expansion. The patient’s neurological deterioration was also likely multifactorial. Severe hypertension at presentation, possible progression of ischemic injury, and hypertensive encephalopathy were important alternative considerations that may have contributed to the altered sensorium. Consequently, hydrocephalus was interpreted as a potentially significant contributing factor. Only a limited number of reports in the literature have described unilateral ventricular enlargement or suspected obstructive hydrocephalus occurring in association with ischemic stroke. Similar reports emphasize the diagnostic difficulty in distinguishing true CSF flow obstruction from ex vacuo ventricular enlargement in patients with chronic infarcts and gliosis. The overlap between symptoms of stroke progression and hydrocephalus may further delay recognition. This case highlights the importance of careful radiologic assessment and consideration of altered CSF dynamics in patients with recurrent stroke who develop neurological deterioration that appears disproportionate to the expected course of ischemic injury.

The management of unilateral hydrocephalus depends on the severity of symptoms and the underlying mechanism of obstruction. While conservative measures such as Head elevation, osmotic agents (e.g., mannitol, hypertonic saline) may be attempted in mild cases. Definitive treatment typically requires surgical intervention to restore CSF flow [8]. Options include endoscopic third ventriculostomy (ETV), which creates an alternative pathway for CSF circulation, and ventriculoperitoneal (VP) shunting, which diverts CSF to the peritoneal cavity. External ventricular drain (EVD), targeted endoscopic procedures, Foraminoplasty of the foramen of Munro, and adhesiolysis of the gliotic obstruction. The choice of procedure depends on multiple factors, including the site of obstruction, ventricular anatomy, and patient-specific considerations [9]. Antiplatelet therapy for ischemic stroke, diabetes control, and hypertension control are examples of underlying etiology management that is also necessary. The majority of the evidence for unilateral hydrocephalus owing to ischemic stroke comes from solitary case reports and tiny case series, despite the condition's clinical significance. This emphasizes the necessity of raising doctors' awareness, especially when assessing patients who have had many strokes and exhibit inexplicable neurological decline. In order to prevent misdiagnosis and to guarantee adequate and prompt therapy, it is crucial to recognize this uncommon issue. It is a varied set of illnesses with a range of clinical presentations, etiologies, and prognostic consequences [10].

## Conclusions

Unilateral hydrocephalus is a rare but important complication in patients with recurrent ischemic stroke and may present with unexplained neurological deterioration. Careful neuroimaging assessment is essential to differentiate possible obstructive hydrocephalus from ex vacuo ventricular dilatation secondary to chronic ischemic and gliotic changes. Early recognition of altered CSF dynamics and timely neurosurgical evaluation may facilitate appropriate management and help prevent further neurological decline and long-term neurological impairment.
